# BH4 domain peptides derived from Bcl-2/Bcl-XL as novel tools against acute pancreatitis

**DOI:** 10.1038/s41420-018-0054-5

**Published:** 2018-05-10

**Authors:** Tim Vervliet, Julia V. Gerasimenko, Pawel E. Ferdek, Monika A. Jakubowska, Ole H. Petersen, Oleg V. Gerasimenko, Geert Bultynck

**Affiliations:** 1 Department of Cellular and Molecular Medicine, Laboratory of Molecular and Cellular Signaling, KU Leuven, Leuven, 3000 Belgium; 2 Medical Research Council Group, Cardiff School of Biosciences, Cardiff University, Cardiff, CF10 3AX UK

## Abstract

Biliary acute pancreatitis (AP) is a serious condition, which currently has no specific treatment. Taurolithocholic acid 3-sulfate (TLC-S) is one of the most potent bile acids causing cytosolic Ca^2+^ overload in pancreatic acinar cells (PACs), which results in premature activation of digestive enzymes and necrosis, hallmarks of AP. The inositol 1,4,5-trisphosphate receptor (IP_3_R) and the ryanodine receptor (RyR) play major roles in intracellular Ca^2+^ signaling. Inhibition of these endoplasmic reticulum-located channels suppresses TLC-S-induced Ca^2+^ release and necrosis, decreasing the severity of AP. Anti-apoptotic B-cell lymphoma (Bcl)-2-family members, such as Bcl-2 and Bcl-X_L_, have emerged as important modulators of IP_3_Rs and RyRs. These proteins contain four Bcl-2 homology (BH) domains of which the N-terminal BH4 domain exerts critical roles in regulating intracellular Ca^2+^ release channels. The BH4 domain of Bcl-2, but not of Bcl-X_L_, binds to and inhibits IP_3_Rs, whereas both BH4 domains inhibit RyRs. Although clear cytoprotective effects have been reported for these BH4 domains, it remains unclear whether they are capable of inhibiting pathological Ca^2+^-overload, associated with AP. Here we demonstrate in PACs that the BH4 domains of Bcl-2 and Bcl-X_L_ inhibit RyR activity in response to the physiological agonist cholecystokinin. In addition, these BH4 domains inhibit pathophysiological TLC-S-induced Ca^2+^ overload in PACs via RyR inhibition, which in turn protects these cells from TLC-S-induced necrosis. This study shows for the first time the therapeutic potential of BH4 domain function by inhibiting pathological RyR-mediated Ca^2+^ release and necrosis, events that trigger AP.

## Introduction

The anti-apoptotic B-cell lymphoma 2 (Bcl-2)-family members, like Bcl-2 and Bcl-X_L_, are critically involved in maintaining mitochondrial integrity by scavenging and inhibiting pro-apoptotic Bcl-2-family members, such as Bax and Bak^1^. This interaction occurs via the hydrophobic cleft, formed by the Bcl-2 homology (BH) domain 1, 2, and 3, of anti-apoptotic Bcl-2 proteins and the BH3 domain of the pro-apoptotic family members. In addition to the hydrophobic cleft the last most N-terminal BH domain, the BH4 domain, is also critical for the anti-apoptotic properties of Bcl-2^2–4^.

Besides neutralizing pro-apoptotic Bcl-2-family members, anti-apoptotic Bcl-2 proteins also emerged as critical modulators of intracellular Ca^2+^ signaling^5–7^. In particular, Bcl-2 is present at the membranes of the endoplasmic reticulum (ER), the main intracellular Ca^2+^-store^8,9^. At the ER, Bcl-2 directly inhibits the inositol 1,4,5-trisphosphate (IP_3_) receptor (IP_3_R)^3,4^, a ubiquitously expressed IP_3_-gated ER-located Ca^2+^ release channel^10^. Bcl-2 forms a protein complex with the IP_3_R by targeting the central modulatory domain of the channel. As such, Bcl-2 limits excessive Ca^2+^-release that may trigger apoptotic cell death. Importantly, the BH4 domain of Bcl-2 by itself is sufficient for inhibiting IP_3_R-mediated Ca^2+^ release, protecting cells against Ca^2+^-induced mitochondrial outer membrane permeabilisation (MOMP) and subsequent apoptosis^3^. Although, the BH4 domain of Bcl-2 and that of Bcl-X_L_ are very similar in size, sequence and structure, the latter is unable to bind to and inhibit IP_3_R-mediated Ca^2+^ release^2^. The difference in IP_3_R-inhibitory properties between the BH4 domain of Bcl-2 and Bcl-X_L_ could be largely attributed to a single amino acid change. Substituting Lys17 for an Asp residue in the BH4 domain of Bcl-2 abrogated its IP_3_R-inhibitory function, while changing Asp11 into a Lys residue in BH4 domain of Bcl-X_L_ enhanced its IP_3_R-inhibitory function^2^.

In addition to the IP_3_R, we recently showed that anti-apoptotic Bcl-2 proteins also bind to and inhibit ryanodine receptors (RyR)^7,11^. RyRs form a second class of the ER-located Ca^2+^ release channels, mainly expressed in specialized tissues such as the heart, brain, muscle, but also in the pancreas^12,13^. In these tissues, RyR-mediated Ca^2+^ release is involved in muscle contraction, memory formation and secretion of digestive enzymes^13,14^. Endogenous activation of RyRs occurs via Ca^2+^-induced Ca^2+^ release or via NAADP and/or cyclic-ADP ribose production^15^. The interaction between RyRs and Bcl-2 is mediated by the BH4 domain of the latter. Binding of the BH4 domain of Bcl-2 to the RyR is sufficient for inhibiting RyR-mediated Ca^2+^ release^7^. In contrast to what was shown for the IP_3_R, the BH4 domain of Bcl-X_L_ also binds to and inhibits RyRs^11^. In our previous work, we demonstrated that Bcl-2 and Bcl-X_L_ (via their BH4 domains) could modulate pharmacological activation of RyRs by caffeine^7,11^. However, prior to the present study it was unclear whether RyR responses to physiological agonists can also be regulated by the BH4 domains.

Furthermore, excessive RyR-mediated Ca^2+^ release is linked to several pathologies and is a hallmark for the onset of several diseases of the brain, heart, muscle, and pancreas, such as Alzheimer’s disease, catecholaminergic polymorphic ventricular tachycardia, malignant hyperthermia, and acute pancreatitis (AP), respectively^13,16^. The BH4 domains of Bcl-2 proteins, particularly Bcl-X_L_, have been shown to have protective anti-apoptotic effects in several of these tissues^17–21^. However, a link between cell protection and RyR inhibition by these BH4 domains has not yet been reported. Therefore, it remains unknown whether the BH4 domains of Bcl-2 and Bcl-X_L_ could be exploited as inhibitors of RyRs in diseases associated with excessive RyR activity in order to dampen disease burden.

To address this, we have chosen pancreatic acinar cells (PACs), in which physiological and pathophysiological intracellular Ca^2+^ signaling has been extensively studied^22–26^. Importantly, in PACs physiologically relevant agonists can be utilized to trigger IP_3_R or RyR-dependent Ca^2+^ releases, allowing to compare the effects of the BH4 domain peptides on both channels^26^. Exposing PACs to low nanomolar concentrations of acetylcholine (ACh) is known to trigger primarily IP_3_R-mediated Ca^2+^ oscillations via activation of phospholipase C leading to IP_3_ production. In contrast, cholecystokinin (CCK) mainly triggers RyR-mediated Ca^2+^ release via the production of NAADP and/or cyclic-ADP ribose^26^. Finally, bile acids, such as taurolithocholic acid 3-sulfate (TLC-S), induce IP_3_R and RyR-mediated Ca^2+^ overload^22^, leading to premature activation of digestive enzymes and subsequent necrosis, which is an initiating event for AP^26^. It has been demonstrated that noxious Ca^2+^ signals induced by TLC-S can be reduced by inhibition of IP_3_Rs or RyRs, using caffeine or dantrolene, respectively^24,25^. Importantly, these approaches not only protected PACs against necrosis but also ameliorated the severity of AP in mouse models. Although it is well known that intracellular Ca^2+^ signaling plays important roles in the development of AP, currently no effective treatment exists for this disease. Here, we test whether the BH4 domains of Bcl-2/Bcl-X_L_ proteins can be utilized to suppress IP_3_R or RyR hyperactivity associated with the onset of AP.

In this study, by employing primary isolated mouse PACs, we show that peptides derived from the BH4 domains of Bcl-2 and Bcl-X_L_ inhibit both physiological and pathophysiological RyR-mediated Ca^2+^ release, as well as protect PACs from TLC-S-induced necrosis. These findings show for the first time that the BH4 domains of Bcl-2 and Bcl-X_L_ can be applied as innovative peptide tools to limit excessive RyR-mediated Ca^2+^ release associated with the pathology of AP. As such, BH4-domain-based molecules and mimetics may originate a novel group of therapeutics with the potential application in diseases associated with excessive Ca^2+^ release.

## Results

### The BH4 domains of Bcl-2 and Bcl-X_L_ inhibit RyR-mediated Ca^2+^ release in PACs

**Fig. 1:**
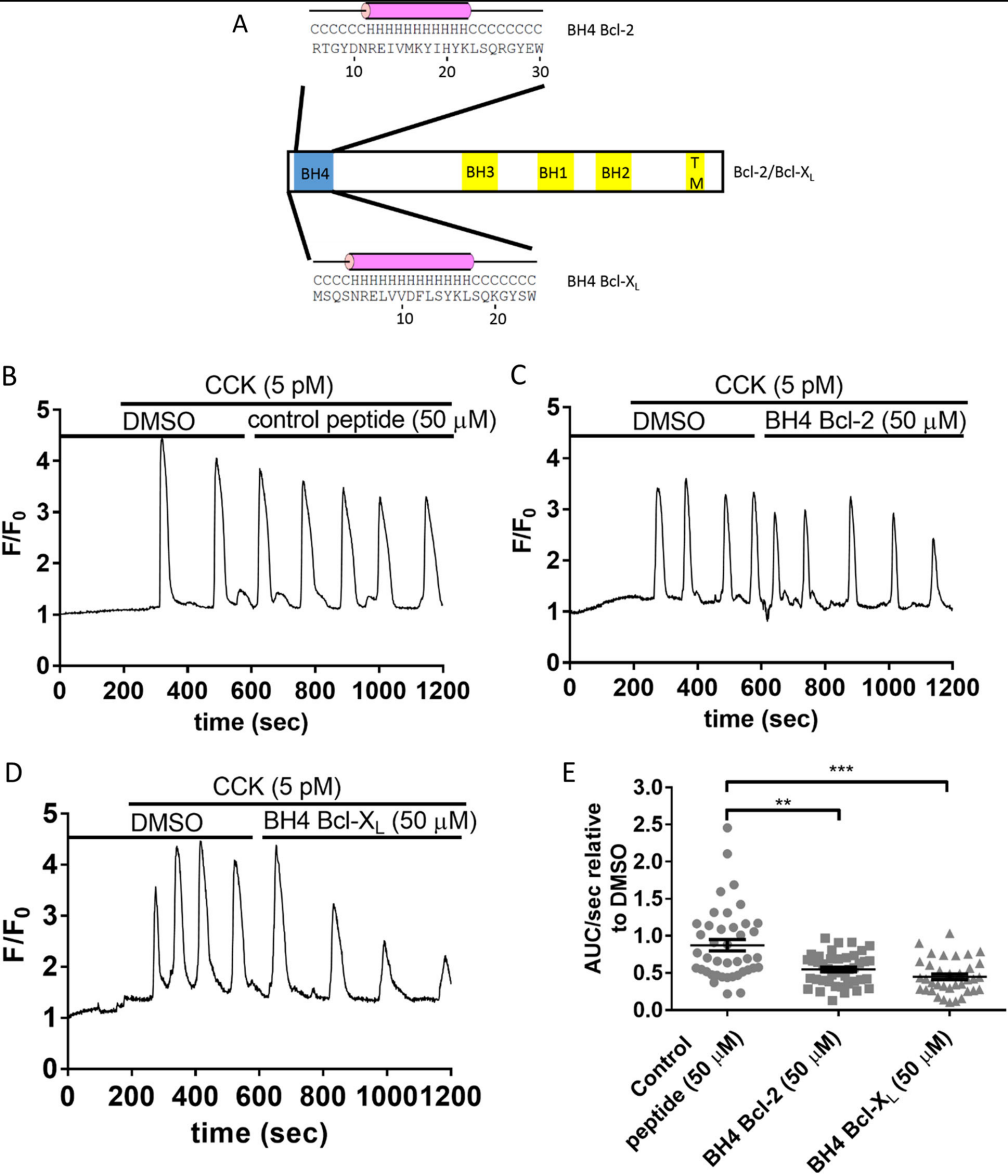
The BH4 domains of Bcl-2 and Bcl-X_L_ inhibit RyR-mediated Ca^2+^ release in PACs. **a** Linear representation of the Bcl-2/Bcl-X_L_ protein together with the sequences used to design the BH4 domain peptides originating from Bcl-2 and Bcl-X_L_ and the predicted secondary structure using the PSIPRED secondary structure predictor (http://bioinf.cs.ucl.ac.uk/psipred/). **b**–**d** Typical traces of the Fluo-4AM single-cell Ca^2+^ measurements performed in mouse PACs. At the start of each experiment cells were perfused with NaHEPES containing DMSO (vehicle). After establishing the baseline (200 s), CCK (5 pM) was added and RyR-mediated Ca^2+^ oscillations were measured for 5 min. Then, 50 µM of either the control peptide (**b**), the BH4 domain of Bcl-2 (**c**), or the BH4 domain of Bcl-X_L_ (**d**) were added for 10 min, in the continuous presence of 5 pM CCK. **e** Quantification of the experiments: The area under the curve per second (AUC/sec) of the CCK-induced Ca^2+^ release after peptide addition (10 min recording) was compared to the AUC/sec before peptide addition (5 min recording). Each data point represents the measurement of an individual cell. The average values ± SEM are shown (*P*-value <0.0001). At least three independent experiments were performed per condition (*N* ≥ 3). For each condition at least 35 cells were recorded

In the first set of experiments, we aimed to assess the ability of the BH4 domains of Bcl-2 and Bcl-X_L_ to inhibit IP_3_R or RyR-mediated Ca^2+^ release in isolated PACs. IP_3_R or RyR-mediated Ca^2+^ release was triggered by the physiological activators ACh or CCK, respectively. The sequences used to design the peptides corresponding to the BH4 domain of Bcl-2 and Bcl-X_L_ with their predicted α-helical properties is depicted in Fig. 1a. In PACs, low nanomolar concentrations of ACh and low picomolar concentrations of CCK generate long lasting Ca^2+^ oscillations, which vary in terms of amplitude and frequency between the cells. Therefore, in single PAC, we compared and quantified the Ca^2+^ oscillations induced by each agonist before and after addition of the BH4 domain peptides or a control peptide. We first assessed the effects of the BH4 domains of Bcl-2 and Bcl-X_L_ on CCK-induced-RyR-mediated Ca^2+^ release (representative traces shown in Fig. 1b–d). Ca^2+^ oscillations were evoked by 5 pM CCK and recorded for 5 min. Then 50 µM control peptide (Fig. 1b), the BH4 domain of Bcl-2 (Fig. 1c) or the BH4 domain of Bcl-X_L_ (Fig. 1d) was added and Ca^2+^ responses were measured for another 10 min, in the continuous presence of CCK. Quantitative analysis of the responses was performed by comparing the area under the curve (AUC). These values were further divided by the length of the recording before (5 min) and after (10 min) addition of the peptides, resulting in the response area normalized per unit of time (AUC/sec). These experiments show that the BH4 domains of Bcl-2 and Bcl-X_L_ dampen RyR-mediated Ca^2+^ oscillations evoked by the physiological stimulus CCK (Fig. 1e) and are in line with our previous findings in dissociated hippocampal neurons and RyR-overexpression models^7,11^. In addition, this also indicates that the BH4 domain peptides are taken up by the PACs and are capable of inhibiting physiological RyR-mediated Ca^2+^ release.

### The BH4 domain of Bcl-2 does not inhibit IP_3_R-induced Ca^2+^release in PACs

**Fig. 2:**
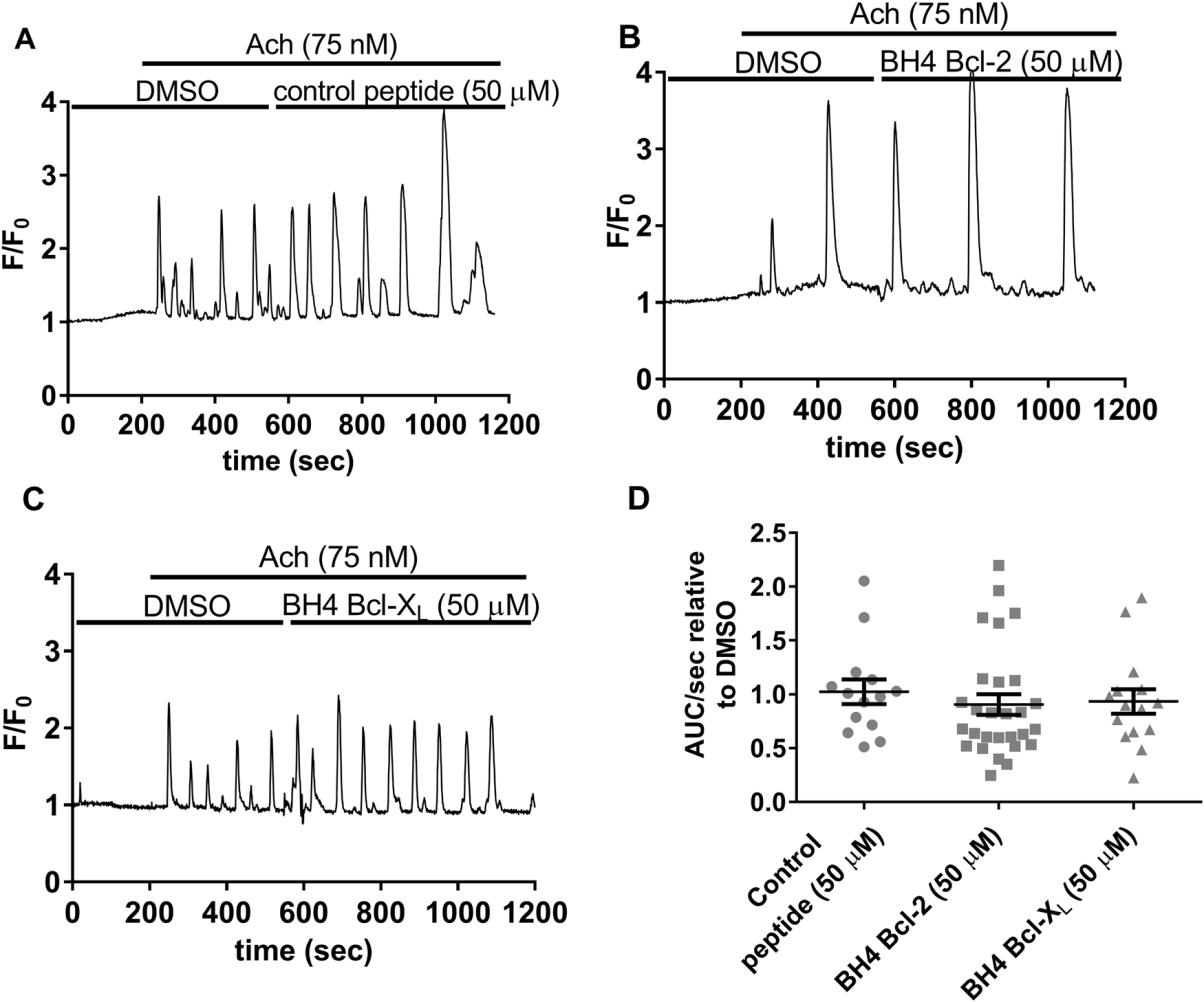
The BH4 domain of Bcl-2 does not inhibit IP_3_R-induced Ca^2+^ release in PACs. **a**–**c** Typical traces of the Fluo-4AM single-cell Ca^2+^ measurements performed in mouse PACs. At the start of each experiment cells were perfused with NaHEPES containing DMSO (vehicle). After establishing the baseline (200 s), ACh (75 nM) was added and IP_3_R-mediated oscillations were measured for 5 min. Then, 50 µM of either the control peptide (**a**), the BH4 domain of Bcl-2 (**b**), or the BH4 domain of Bcl-X_L_ (**c**) were added for 10 min, in the continuous presence of 75 nM ACh. **d** Quantification of the experiments: The area under the curve per second (AUC/sec) of the ACh-induced Ca^2+^ release after peptide addition (10 min recording) was compared to the AUC/sec before peptide addition (5 min recording). Each data point represents the measurement of an individual cell. The average values ± SEM are shown (*P*-value 0.3467). At least three independent experiments were performed per condition (*N* ≥ 3). For each condition at least 14 cells were recorded

Next, we assessed the effects of the BH4 domain peptides on IP_3_R-mediated Ca^2+^ release. Analogical to CCK experiments single-cell Ca^2+^ measurements were performed using ACh (75 nM) in order to trigger IP_3_R-mediated Ca^2+^ oscillations (Fig. 2). As expected, subsequent addition of the control peptide or the BH4 domain of Bcl-X_L_ did not result in an inhibition of ACh-induced-IP_3_R-mediated Ca^2+^ release (Fig. 2a, c, d). Surprisingly, also the BH4 domain of Bcl-2 was unable to inhibit IP_3_R-mediated Ca^2+^ release in PACs (Fig. 2b, d). These results differ compared to what we showed previously in cultured or permeabilized cells where the BH4 domain of Bcl-2 inhibited IP_3_R-mediated Ca^2+^ release via its BH4 domain^4,27–29^.

### The BH4 domain of Bcl-2 inhibits IP_3_R-mediated Ca^2+^ release in Bcl-2 knock out PACs

The lack of inhibition of IP_3_R-mediated Ca^2+^ release upon application of the BH4 domain of Bcl-2 could be due to the fact that endogenous Bcl-2 in wild-type (WT) PACs is already associated with IP_3_Rs, preventing its regulation by exogenously added BH4 domain of Bcl-2. To address this, we performed single-cell Ca^2+^ measurements in PACs isolated from Bcl-2 knock out (KO) mice, devoid of endogenous Bcl-2.

**Fig. 3:**
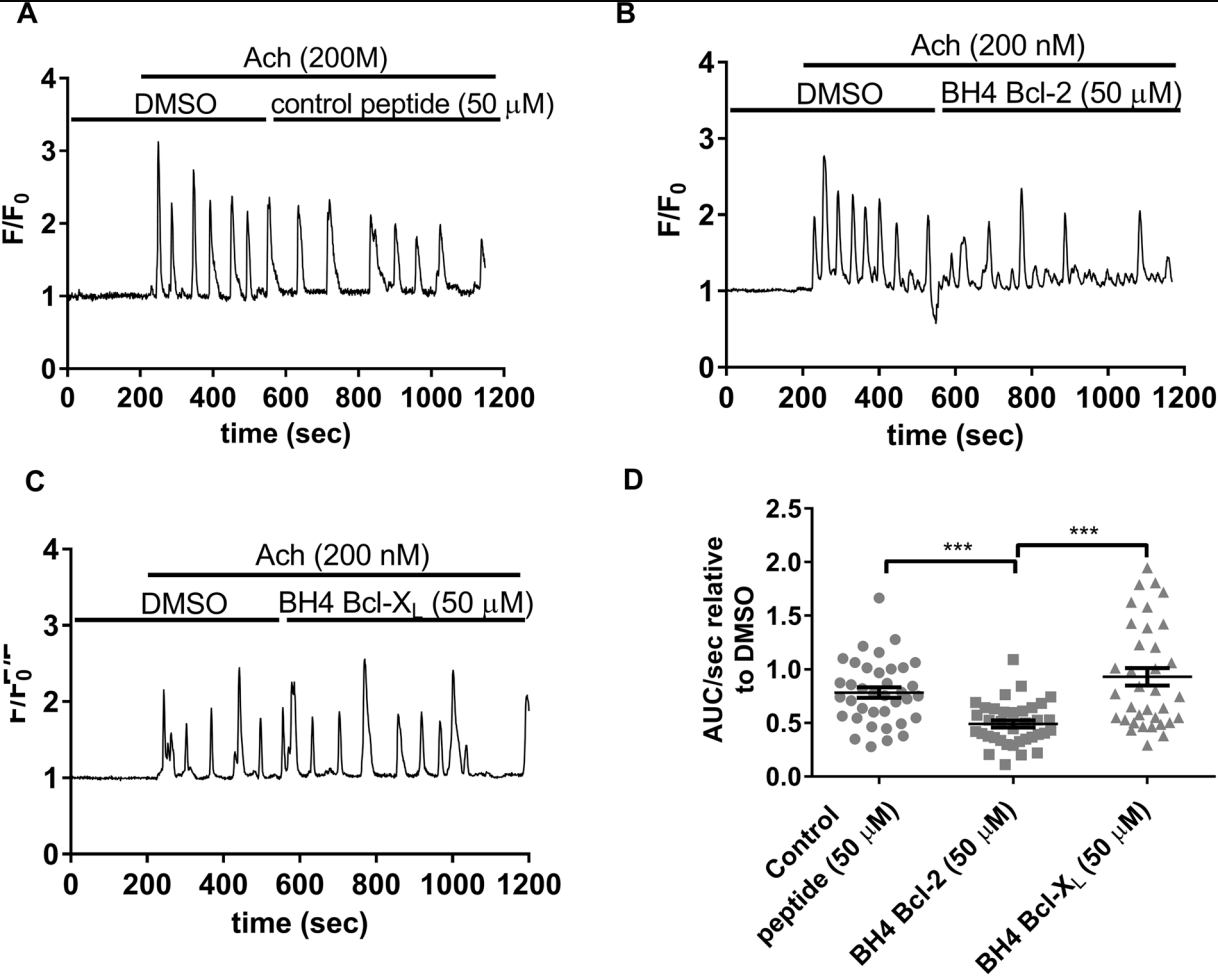
The BH4 domain of Bcl-2 inhibits IP_3_R-mediated Ca^2+^ release in Bcl-2 KO PACs. **a**–**c** Typical traces of the Fluo-4AM single-cell Ca^2+^ measurements performed in Bcl-2 KO mouse PACs. At the start of each experiment cells were perfused with NaHEPES containing DMSO (vehicle, Fig. 3a). After establishing the baseline (200 s), ACh (200 nM) was added and IP_3_R-mediated Ca^2+^ oscillations were measured for 5 min. Then, 50 µM of either the control peptide (**a**), the BH4 domain of Bcl-2 (**b**) or the BH4 domain of Bcl-X_L_ (**c**) were added for 10 min, in the continuous presence of 200 nM ACh. **d** Quantification of the experiments: The area under the curve per second (AUC/sec) of the ACh-induced Ca^2+^ release after peptide addition (10 min recording) was compared to the AUC/sec before peptide addition (5 min recording). Each data point represents the measurement of an individual cell. The average values ± SEM are shown (*P*-value <0.0001). At least three independent experiments were performed per condition (*N* ≥ 3). For each condition at least 35 cells were recorded

In order to obtain repeatable, long lasting Ca^2+^ oscillations in Bcl-2 KO PACs, similar in frequency and amplitude to those evoked in WT PACs, high-nanomolar concentrations (200 nM) of ACh were required. Consistent with our previous findings, addition of 50 µM control peptide or the BH4 domain of Bcl-X_L_ (Fig. 3a, c, d) did not alter IP_3_R-mediated Ca^2+^ releases. However, in Bcl-2 KO PACs, the BH4 domain of Bcl-2 does significantly inhibit ACh-induced-IP_3_R-mediated Ca^2+^ release (Fig. 3b, d) compared to control peptide or the BH4 domain of Bcl-X_L_.

**Fig. 4:**
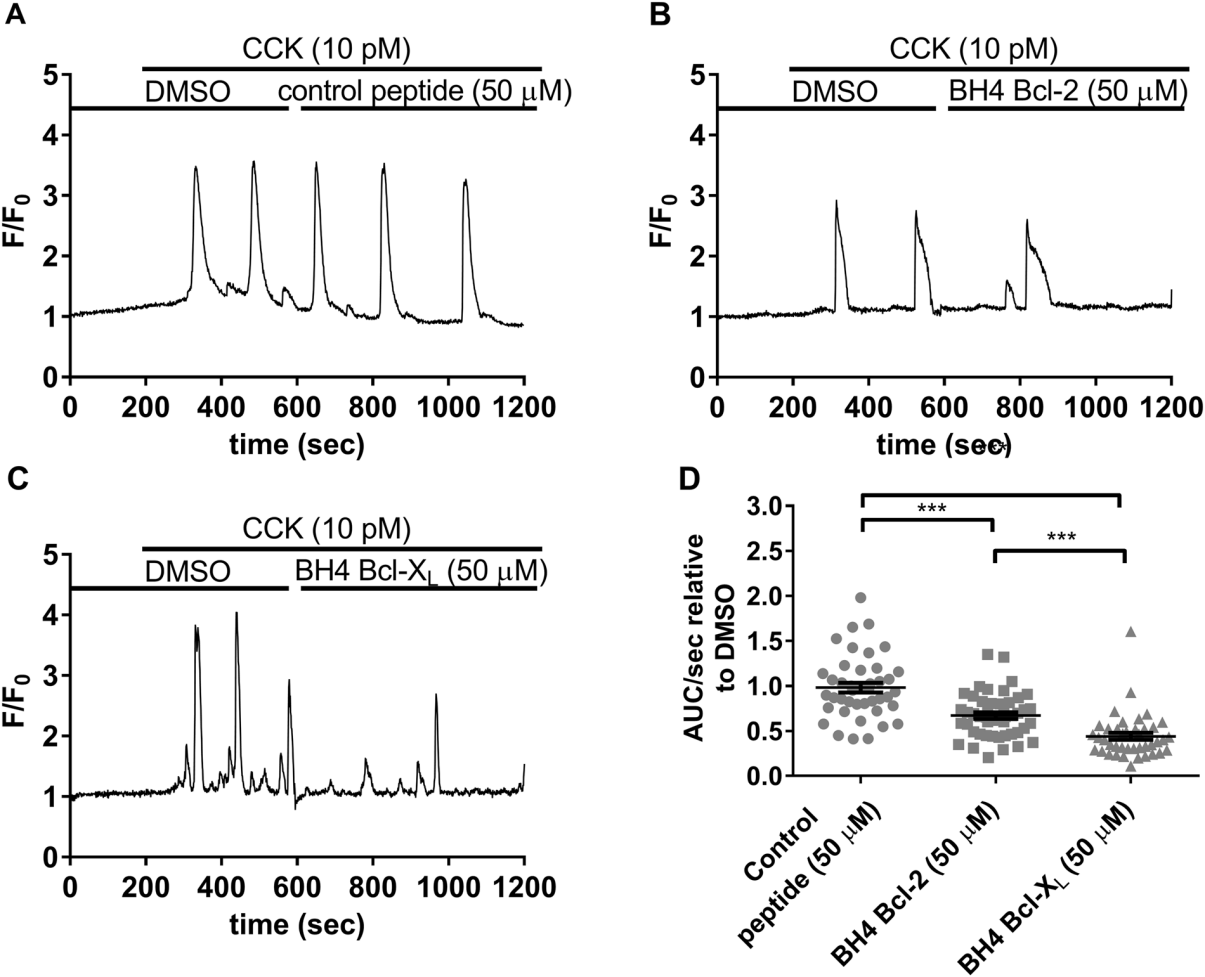
The BH4 domains of Bcl-2 and Bcl-X_L_ inhibit RyR-mediated Ca^2+^ release in Bcl-2 KO PACs. **a**–**c** Typical traces of the Fluo-4AM single-cell Ca^2+^ measurements performed in Bcl-2 KO mouse PACs. At the start of each experiment cells were perfused with NaHEPES containing DMSO (vehicle). After establishing the baseline (200 s), CCK (10 pM) was added and RyR-mediated Ca^2+^ oscillations were measured for 5 min. Then, 50 µM of either the control peptide (**a**), the BH4 domain of Bcl-2 (**b**), or the BH4 domain of Bcl-X_L_ (**c**) were added for 10 min, in the continuous presence of 10 pM CCK. **d** Quantification of the experiments: The area under the curve per second (AUC/sec) of the CCK-induced Ca^2+^ release after peptide addition (10 min recording) was compared to the AUC/sec before peptide addition (5 min recording). Each data point represents the measurement of an individual cell. The average values ± SEM are shown (*P*-value <0.0001). At least three independent experiments were performed per condition (*N* ≥ 3). For each condition at least 40 cells were recorded

The inhibitory properties of the BH4 domain peptides were also tested on CCK-evoked oscillations in Bcl-2 KO PACs (Fig. 4a–d). In the Bcl-2 KO PACs, both the BH4 domains of Bcl-2 and Bcl-X_L_ showed a similar response inhibition (±50% AUC/sec) as seen in the WT PACs. Comparing WT to Bcl-2 KO PACs (Figs. 1, 4) shows that the BH4 domains did not confer additional inhibition of CCK-induced Ca^2+^ release in the absence of Bcl-2. Taken together these functional experiments suggest that in WT PACs endogenous Bcl-2 mainly occupies/regulates IP_3_Rs and to a lesser extent RyRs. This potentially leaves more RyR channels available for binding to exogenously added BH4 domain of Bcl-2, resulting in efficient inhibition of RyRs but not IP_3_Rs in WT PACs. Of note, just like in the experiments utilizing ACh in Bcl-2 KO PACs (Fig. 3), higher concentrations of CCK (10 pM) were needed to trigger RyR-mediated Ca^2+^ oscillations of comparable frequency and amplitude to those recorded in WT PACs (Fig. 4).

### The BH4 domains of Bcl-2 and Bcl-X_L_ inhibit pathological TLC-S induced Ca^2+^ releases in PACs

Combining the above results from ACh and CCK-induced Ca^2+^ release, we can conclude that in WT PACs the inhibitory effects of the BH4 domains of Bcl-2 and Bcl-X_L_ on Ca^2+^ release from the ER is due to the inhibition of RyR- but not IP_3_R-mediated Ca^2+^ release. This has prompted us to investigate whether the BH4 domains of Bcl-2 and Bcl-X_L_ could also suppress pathological RyR-mediated Ca^2+^ releases.

**Fig. 5:**
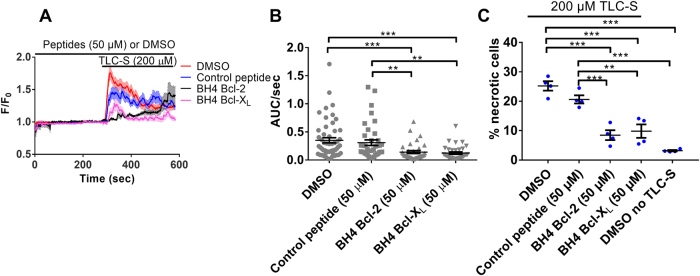
The BH4 domains of Bcl-2 and Bcl-X_L_ inhibit pathological TLC-S-induced Ca^2+^ release and necrosis in PACs by inhibiting RyR activity. **a** Average traces of Fluo-4AM single-cell Ca^2+^ measurements performed in PACs. The cells were pre-treated for 5 min with either DMSO (vehicle; red) or 50 µM of the peptides: control (blue), the BH4 domain of Bcl-2 (black), and the BH4 domain of Bcl-X_L_ (pink). Then, TLC-S (200 µM) was added and pathological intracellular Ca^2+^ release was measured for 5 min, in the continuous presence of either vehicle or the peptides. Average traces ± SEM of all performed experiments are shown. **b** Quantification of the experiments: Analysis of area under the curve per second (AUC/sec) of the TLC-S-induced Ca^2+^ releases, in the presence or absence of the BH4 domains or control peptide. Each data point represents the measurement of an individual cell, and the average values ± SEM are shown (*P*-value <0.0001). At least three independent experiments were performed per condition (*N* ≥ 3). For each condition at least 35 cells were recorded. **c** Quantification of the necrosis assay: Isolated PACs were treated with DMSO (vehicle) or 50 µM of the indicated peptides. 15 min later, TLC-S was added (200 µM final concentration) to induce necrotic cell death. Propidium iodide staining (necrosis indicator) was assessed 2 h after TLC-S addition. The negative control was treated with DMSO (vehicle) only. In each experimental repeat (*N* = 4) at least 15 images were taken per treatment group (*n* ≥ 100 cells/treatment/experiment). The percentages of propidium iodide-positive necrotic cells were assessed for each experimental condition. Each data point represents an independent repeat of the experiment, and the average values ± SEM are shown (*P*-values <0.001)

The bile acid TLC-S has been described to mediate excessive, pathological intracellular Ca^2+^ release in PACs and both IP_3_Rs and RyRs have been proposed to be involved in this process^22^. Here we aimed to test whether the BH4 domains of Bcl-2 and Bcl-X_L_ could also inhibit pathophysiological TLC-S-induced Ca^2+^ release (Fig. 5). WT PACs were pre-treated for 5 min with either DMSO (vehicle) or 50 µM of the peptides, then TLC-S (200 µM) was added and Ca^2+^ responses were measured (Fig. 5a). Detailed comparison of the response areas recorded for the individual cells (AUC) per unit of time (AUC/sec) revealed that both BH4 domains inhibited pathological TLC-S-induced Ca^2+^ responses compared to the control peptide and DMSO vehicle (Fig. 5b). Both BH4 domains inhibit RyR but not IP_3_R-mediated Ca^2+^ release (Figs. 1, 2) and similarly suppress TLC-S-induced Ca^2+^ release in WT PACs (Fig. 5a, b). Collectively, these data indicate that the BH4 domains suppress pathophysiological Ca^2+^ signaling by acting as modulators of the excessive RyR activity.

### The BH4 domains of Bcl-2 and Bcl-X_L_ inhibit TLC-S-induced necrosis in PACs by inhibiting excessive RyR activity

It has been well established that TLC-S-induced Ca^2+^ release in PACs leads to necrosis which is an underlying cause of AP^23^. In addition, pharmacological inhibition of either RyRs or IP_3_Rs, using dantrolene or caffeine, respectively, not only attenuated TLC-S-induced necrosis, but also reduced the severity of AP evoked in mouse models of this disease^24,25^.

Since the BH4 domains of Bcl-2 and Bcl-X_L_ proved to be the effective inhibitors of TLC-S-induced Ca^2+^ release via inhibiting RyRs (Fig. 5a, b), we aimed to determine whether these peptides could protect PACs against the cytotoxic effects of TLC-S. In the PACs, 50 µM BH4 domain of Bcl-2 or Bcl-X_L_ almost completely inhibited necrotic cell death induced by 2 h treatment with 200 µM TLC-S, compared to the control peptide or the vehicle control (Fig. 5c and S1). This demonstrates a proof-of-principle that the BH4 domains of Bcl-2 and Bcl-X_L_ could be applied as a basis for developing therapeutic tools to decrease the necrotic burden of bile acids on PACs by preventing the excessive RyR-mediated Ca^2+^ release.

## Discussion

The main conclusions of this paper are that (i) the BH4 domains of Bcl-2 and Bcl-X_L_ suppress physiological RyR-mediated Ca^2+^ release in isolated PACs exposed to the hormone CCK; (ii) the Ca^2+^-signaling-inhibitory properties of these BH4 domains can be utilized to dampen pathophysiological RyR-mediated cytosolic Ca^2+^ overload associated with AP, protecting PACs against necrosis. Furthermore, these results indicate for the first time a potential for the therapeutic application of BH4 domains, or therapeutic tools derived from them, as suppressors of (excessive) RyR-mediated Ca^2+^ release in the treatment of AP.

In our previous work, we have already shown that Bcl-2, Bcl-X_L_ and the BH4 domains of these proteins inhibit RyR-mediated Ca^2+^ release^7,11^. In those studies, RyR-overexpression models and dissociated hippocampal neurons were investigated and RyR activation was attained pharmacologically using caffeine. In this study, we provide evidence that the BH4 domains of Bcl-2 and Bcl-X_L_ inhibit RyR activity triggered by the hormone CCK in a physiologically relevant concentration (5 pM) (Fig. 1). Therefore, in PACs the BH4 domains of Bcl-2 and Bcl-X_L_ may serve as the modulators of RyR-mediated Ca^2+^ signals.

The lack of inhibition of IP_3_R-mediated Ca^2+^ release in WT PACs by the BH4 domain of Bcl-2 was somewhat surprising (Fig. 2). However, this might be due to several factors. First, in most of the previous studies, the peptides were either loaded into the cells via electroporation or were added to already permeabilized cells^2,4,28^. Here, the BH4 domain peptides were added directly to intact cells without any adjuvant approach. This, however, could limit the intracellular concentration of the peptides and prevent it from reaching the levels required for inhibiting IP_3_Rs. Another possible explanation of these findings may be related to the proteolytic activation of the digestive enzymes and degradation of the BH4 domain of Bcl-2 or Bcl-X_L_ into smaller fragments, capable of inhibiting RyRs but not IP_3_Rs. Although our experiments were performed in the presence of an extracellular protease inhibitor (inhibiting trypsin and chymotrypsin), we cannot fully rule out the possibility of enzymatic degradation of the BH4 domains. Finally, we hypothesize that IP_3_Rs in the WT PACs may already be associated with endogenous Bcl-2 and thus application of the BH4 domain of Bcl-2 may not provide any additional inhibition. To investigate this, we performed control experiments using PACs isolated from Bcl-2 KO mice. Importantly, in the cells lacking endogenous Bcl-2, the exogenous BH4 domain of Bcl-2 was capable of inhibiting ACh-induced IP_3_R-mediated Ca^2+^ release (Fig. 3). Also in the Bcl-2 KO PACs, no additional inhibition of CCK-induced-RyR-mediated Ca^2+^ release by either BH4 domain was detected compared to the WT PACs (Figs. 1 and 4), suggesting that in WT PACs RyRs are not heavily regulated by endogenous Bcl-2. Collectively, these results support the hypothesis that in WT PACs endogenous Bcl-2 may be associated mainly with IP_3_Rs, limiting the potential of the exogenously added BH4 domain of Bcl-2 to inhibit IP_3_R-mediated Ca^2+^ release.

We also want to highlight that higher concentrations of ACh (200 nM) or CCK (10 pM) were needed in Bcl-2 KO PACs compared to wild-type PACs to evoke IP_3_R- or RyR-mediated Ca^2+^ oscillations, respectively (Figs. 3 and 4). Since Bcl-2 is an inhibitor of both IP_3_Rs and RyRs, it is anticipated that Bcl-2 KO PACs should be more sensitive to ACh and CCK. It remains unclear whether in Bcl-2 KO PACs this phenomenon occurs due to the compensatory mechanisms or other adaptive processes during pancreatic development. It is possible that lack of Bcl-2 in PACs results in reduced expression of ACh and CCK receptors, declined levels of the proteins involved in generation of IP_3_, NAADP, cADP-ribose, or decreased levels of IP_3_Rs and RyRs. It is also important to note that, compared to the WT mice, the Bcl-2 KO mice suffer from growth retardation, facial malformation and development of polycystic kidney disease^30^. Also, the pancreata of these Bcl-2 KO mice are much smaller compared to the WT pancreata. Thus, this phenotypic difference may account for the apparent reduced responsiveness of Bcl-2 KO mouse PACs towards extracellular agonists.

Bile acids, such as TLC-S, are well known to induce pathological Ca^2+^ release in PACs, leading to extensive tissue necrosis and development of AP^23^. The involvement of both IP_3_R- and RyR-mediated Ca^2+^ release in this process has been previously demonstrated^22^. From our previous work, we already knew that the BH4 domain of Bcl-X_L_ does not inhibit IP_3_R-mediated Ca^2+^ release^2^. In the present study, we confirmed that observation in PACs (Fig. 2). We also demonstrated that the BH4 domain of Bcl-2 inhibits IP_3_R-mediated Ca^2+^ release in Bcl-2 KO but not in WT PACs (Figs. 2, 3). In addition, we show that both the BH4 domains of Bcl-2 and Bcl-X_L_ similarly inhibit pathophysiological Ca^2+^ overload induced by the bile acid TLC-S in WT PACs (Fig. 5a, b). Combining these observations we conclude that, in WT PACs the inhibition of TLC-S-induced Ca^2+^ release conferred by the BH4 domain peptides is mainly due to the inhibition of RyRs and not IP_3_Rs.

Pharmacological inhibition of either IP_3_Rs or RyRs has been shown to reduce TLC-S-induced necrosis in PACs and the severity of AP^24,25^. Here we confirmed this by showing that the BH4 domains were able to inhibit TLC-S induced necrosis (Fig. 5c). These findings underpin the therapeutic properties of Ca^2+^-signaling modulation in AP and add the BH4 domains of Bcl-2 and Bcl-X_L_ to the arsenal of tools with the therapeutic potential to reduce AP burden by limiting the excessive RyR activity and cell necrosis.

Previous studies on the BH4 domains of the Bcl-2-family proteins have revealed their protective properties against a wide range of pathological stimuli^17–21,31–35^, particularly with respect to the regulation of mitochondrial integrity and thus protection from apoptosis^17–21^. However, whether these BH4 domains could also antagonize cell damage by inhibiting excessive intracellular Ca^2+^ release, especially aberrant RyR activity under pathological conditions, has never been addressed. Here we show for the first time that these BH4 domains may provide protection in AP, a disease characterized by Ca^2+^-induced necrosis, via suppressing excessive RyR activity (Fig. 5).

Although, the exact length of the BH4 domains used in different studies may differ slightly, the amino acid stretch conferring the α-helical properties in the BH4 domains (Fig. 1a) is always present^17–21,31–35^. The α-helical properties of the BH4-domain peptide of Bcl-2 were previously shown to be crucial for inhibiting both IP_3_R activity and suppressing apoptosis induction, rendering this an important feature for the biological activity of the BH4 domain of Bcl-2^28^. In the full-length protein, the BH4 domain also contributes to the overall stability of the Bcl-2 proteins^36^. PACs are known to take up small peptides that retain their bio-activity^37^. Hence, in this work, unmodified BH4-domain peptides could be applied, as they were taken up by intact primary PACs and they retained their biological activity (Figs. 1–5). At this point, it remains unclear whether other cells besides PACs are able to accumulate these unmodified peptides and whether they can be applied in vivo. An often used strategy to improve the uptake of peptides by cells is to couple peptides to a cell-penetrating sequence, like TAT, the protein-transduction domain of the HIV-1 TAT protein^38^. Besides enhancing cell uptake, this tag also introduces several positive charges, improving peptide solubility and bio-availability. This approach has already been used for the BH4 domain of Bcl-X_L_. TAT-tagged BH4 domain of Bcl-X_L_ retained its biological activity when injected intravenously or intraperitonealy in mice, protecting hearth cells from ischemia reperfusion-induced cell death^17,21^, rescuing astrocyte degeneration in amyotrophic lateral sclerosis^39^ and protecting neurons from apoptosis upon frataxin depletion by restoring proper Ca^2+^ homeostasis and dynamics^40^. Another option/modification for further investigating the therapeutic potential of these BH4 domains is the use of stapled BH4 domains^41,42^. Stapled BH4 domains are cyclic peptides with increased stability and cell permeability. Such tools may enable to explore the therapeutic potential of BH4-domains of Bcl-2/Bcl-X_L_ proteins and derivatives in in vivo models for AP.

Taken together, we here report that the BH4-domains of Bcl-2-family members can be utilized as peptide tools with Ca^2+^-modulatory properties to reduce disease burden in disorders such as AP. This work reveals that the BH4 domain of Bcl-2 and Bcl-X_L_, or tools mimicking their functions, may prove useful for therapeutic modulation of the pathologies in which excessive intracellular Ca^2+^ release is a critical driver.

## Materials and methods

### Reagents, mouse strains, secondary structure prediction, and peptides

Unless otherwise specified all reagents were obtained from Sigma-Aldrich (Dorset, UK). Transgenic Bcl-2 KO mice (B6;129S2-Bcl-2) were purchased from The Jackson Laboratory. Homozygous Bcl-2 KO and the WT litter mates were used for the experiments. All regulated animal procedures were subject to approval by the University’s Animal Welfare and Ethical Review Body (AWERB), and covered by a Project Licence granted by the Home Office under the Animal (Scientific Procedures) Act, 1986. PSIPRED version 3.3 (http://bioinf.cs.ucl.ac.uk/psipred/) was utilized to predict the secondary structure of the BH4 domains of Bcl-2 and Bcl-X_L_.

The following peptides (purity ≥80%) were obtained from LifeTein, validated via mass spectrometric analysis, and dissolved in DMSO (30 mM stock solution).

Control peptide: WYEKQRSLHGIMYYVIEDRNTKGYR

BH4 domain Bcl-2: RTGYDNREIVMKYIHYKLSQRGYEW

BH4 domain Bcl-X_L_: MSQSNRELVVDFLSYKLSQKGYSW

### PAC isolation

Mouse PACs were isolated using a modified protocol described in ref. ^37^. Briefly, the pancreas of WT or Bcl-2 KO mouse was dissected and washed twice in NaHEPES buffer (140 mM NaCl, 4.8 mM KCl, 1 M MgCl_2_, 10 mM HEPES, 10 mM glucose and 1 mM CaCl_2_; pH 7.2). 1 ml collagenase (25 U/ml) (C9263) was injected into the pancreas and then the tissue was incubated at 37 °C in a water bath shaker. After this the pancreas was broken down by several cycles of pipetting in 1 ml NaHEPES buffer. The isolated PACs were spun down (200×*g*, 1 min), washed with NaHEPES and then were suspended in fresh NaHEPES.

### PAC loading and single-cell Ca^2+^ measurements

Isolated PACs were incubated in NaHEPES containing 4 µM Fluo4-AM (Life Technologies, Loughborough, UK) for 30 min at room temperature. Then PACs were spun down and resuspended in NaHEPES. Single-cell Ca^2+^ measurements were performed as indicated in the figure descriptions, using a Scientifica (East Sussex, UK) imaging system connected to an Olympus (Cardiff, UK) IX71 microscope. In order to prevent enzymatic digestion of the peptides, the extracellular buffer was supplemented with 0.2 mg/ml trypsin-chymotrypsin inhibitor. The peptides and indicated stimuli were introduced to the cells using a syringe-driven perfusion system.

### Necrosis assay

Isolated PACs were treated with either vehicle (DMSO) or the indicated peptides (50 µM) for 15 min. Then cell death was induced by adding TLC-S (final concentration: 200 μM) to the PACs for 2 h at room temperature. Propidium iodide (2 µg/ml; Life Technologies) was present during the last 15 min of the TLC-S treatment. For the peptide-treated cells, 0.2 mg/ml trypsin-chymotrypsin inhibitor was added in order to protect the peptides from proteolytic damage. A TCS SPE confocal microscope or a multiphoton SP5 (both from Leica, Milton Keynes, UK) were used to image the propidium iodide staining and cell death was quantified by counting the propidium iodide-positive cells in relation to the control. In each experiment at least 100 cells were imaged and counted per treatment group.

### Statistical analysis

For statistical analysis GraphPad prism 7 was used. Because of lack of normal distribution and unequal variance in several tested groups, the Kruskal–Wallis test with the Dunn’s multiple comparison post hoc test was performed for all Ca^2+^ measurements. One-way ANOVA with the Tukey’s multiple comparison post hoc tests were performed for the necrosis assays. When using the One-way ANOVA, the data were normally distributed and variances were not significantly different between the tested groups. *P*-values for each statistical analysis are given in the figure legends. *, **, *** are used to indicate *P*-values <0.05, 0.01, and 0.001 between the different groups as indicated by the post hoc test. For each experimental setup, PACs were isolated and used independently from at least three different mice.

## Electronic supplementary material

Supplementary figure 1
