# Supplementary material for: BH4 domain peptides derived from Bcl-2/Bcl-XL as novel tools against acute pancreatitis
Source: Cell Death Discov. 2018 May 10;4:58. doi: 10.1038/s41420-018-0054-5 (PMC5945673; doi:10.1038/s41420-018-0054-5)
Supplement: Supplementary file 1 — Supplementary figure 1 [file 41420_2018_54_MOESM1_ESM.pdf]

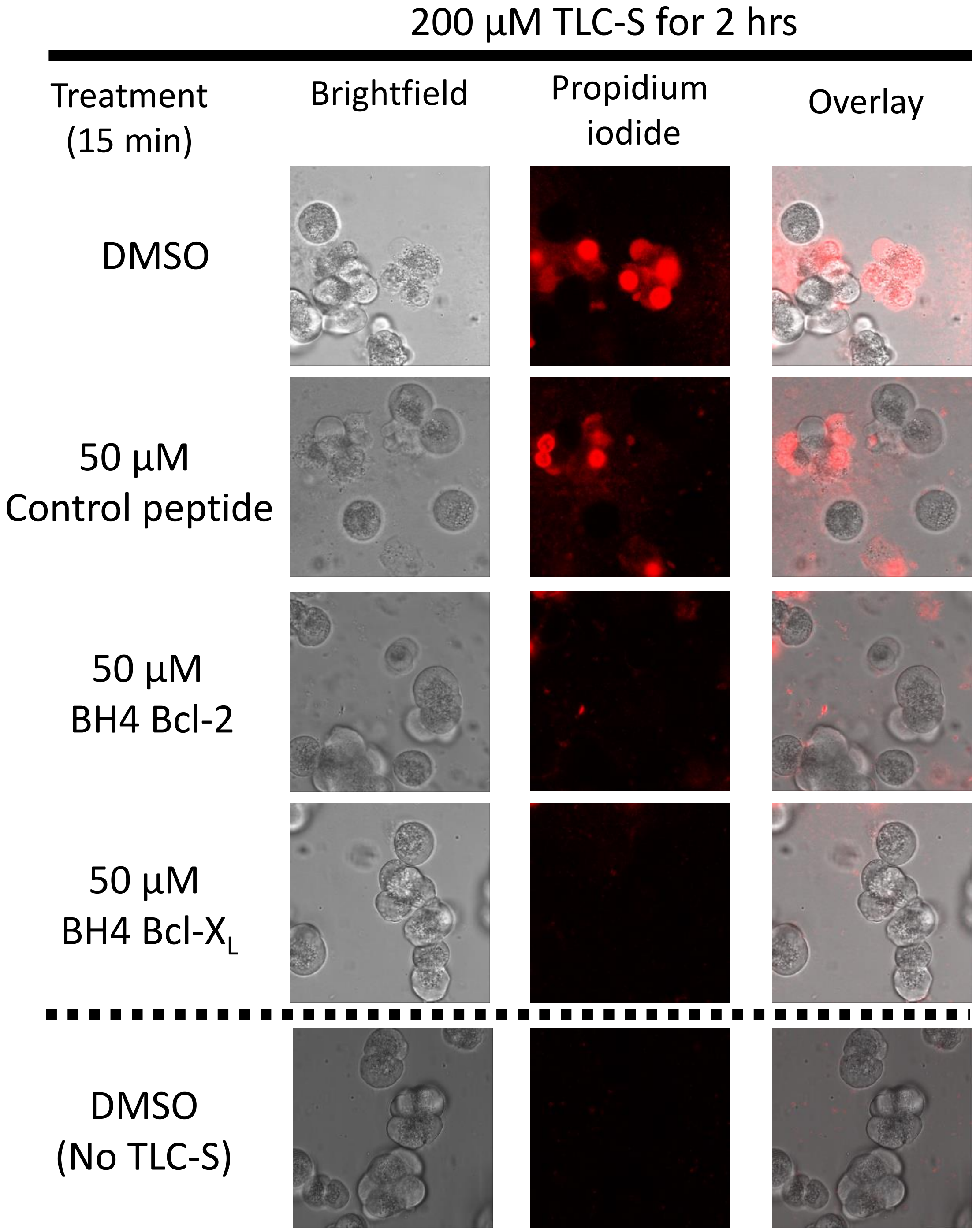

Fig. S1. The BH4 domains of Bcl-2 and Bcl-X_L inhibit TLC-S induced necrosis. Necrosis assay using propidium iodide to identify necrotic PACs isolated from WT mice. Representative images of propidium iodide staining including brightfield and overlay of the performed experiments. Isolated WT PACs were treated with DMSO (vehicle) or 50 μM of the indicated peptides. 15 minutes later TLC-S (200 μM final concentration) was added to induce necrosis. Propidium iodide staining was assessed after 2 hours of TLC-S treatment. The negative control was pre-treated with DMSO but did not receive TLC-S treatment.
